# Exploring the correlates of COVID-19 vaccination inequity: a global analysis using machine learning from a health economic lens

**DOI:** 10.3389/frhs.2026.1774077

**Published:** 2026-05-01

**Authors:** Moumita Mukherjee

**Affiliations:** Institute of International Health, Charité – Universitätsmedizin Berlin, Berlin, Germany

**Keywords:** concentration index (CI), COVID-19, ensemble learning, machine learning, vaccination inequity

## Abstract

**Introduction:**

The COVID-19 pandemic has reduced system resilience, caused economic welfare loss, and widened disparities in healthcare access. This study aims to identify the macro-level factors contributing to COVID-19 vaccination inequity by proposing an AI-driven monitoring framework using classical statistical modelling and machine learning (ML) classifiers from a pooled dataset (WHO, World Bank, and our World in Data) covering 195 countries.

**Methods:**

Daily vaccination data and national-level indicators were investigated using concentration indices, classical odds ratios across global regions and seven ML classifiers—logistic regression, naïve Bayes, decision tree, random forest, LightGBM, extra trees, and XGBoost—to identify significant predictors of vaccination inequity with higher accuracy.

**Results and discussion:**

Findings show vaccine coverage remains pro-rich in Africa, North America, Europe, and Oceania, whereas Southeast Asia depicts pro-poor vaccine uptake. Capacity of health system, availability of hygiene infrastructure, prioritization of people suffering from noncommunicable diseases, and exposure to behavioral risk factors were strongly associated with pro-poor distribution of vaccine access in low-income (LIC) and lower-middle income countries (LMIC) in Asia and Africa and improves over time. Among ML models, random forest and XGBoost achieved the highest performance. As the final best model, random forest (RF) is selected with highest weighted score (98.5%), AUROC (99.9%). XGBoost is the second-best model attained the second highest weighted score (97.9%), AUROC (99.8%), and both attained good 5-fold cross validation standard deviation (0.009 for RF and 0.014 for XGBoost) allowing temporal stratification of folds. Results justify the superiority of ensemble ML models over single learners in predicting inequity in vaccine uptake. This study proves that machine learning outperforms conventional predictive analysis and more suitable for monitoring inequity in COVID-19 vaccination access to inform global health policy towards intelligent pandemic preparedness. Therefore, to reduce regional inequity in vaccine uptake, the regional-structural nonlinearities in LICs and LMICs should be adjusted through accurate and robust integration of artificial intelligence in monitoring systems.

## Introduction

**Figure 1:**
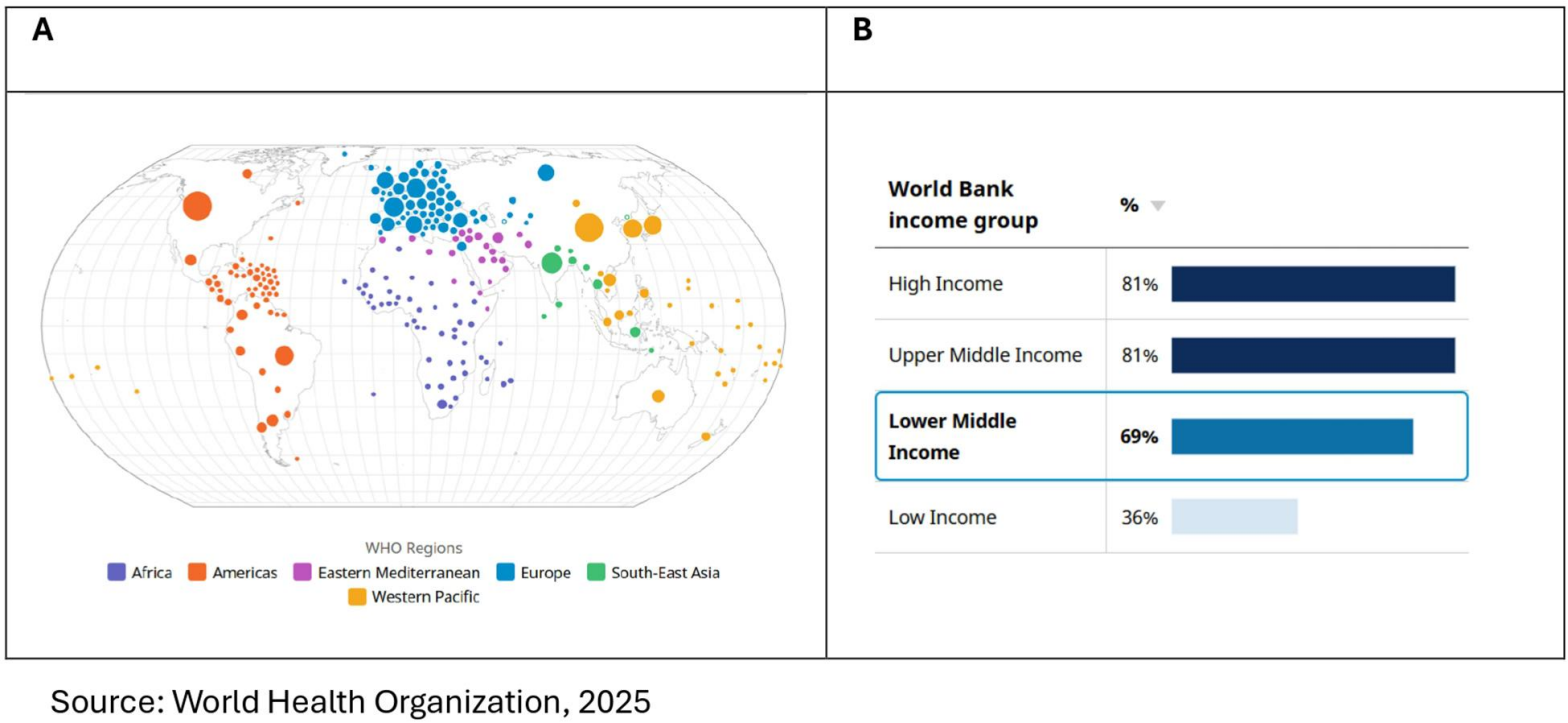
**(A)** Number of COVID-19 cases reported to WHO (cumulative total) in WHO regions [source: WHO dashboard, (4)] and **(B)** percentage of total population vaccinated with at least one dose of a COVID-19 vaccine in countries by World Bank income group, 31 December 2023.

The COVID-19 pandemic disrupted the global health service delivery system, urging for revisiting pandemic preparedness and health system readiness to adopt contextual innovation (1–3). According to the World Health Organization (WHO) factsheet, 760 million confirmed cases and 6.9 million deaths from COVID-19 have been reported globally since December 2019 (4). In response to the pandemic, more than 13 billion vaccine doses are administered until June 2023, and 14.4 million deaths are averted globally after the vaccine rollout (4–6). Furthermore, evidence indicates that every 6 out of 100 people who have recovered from COVID-19 have developed post-COVID-19 health conditions (4). Disaggregated data by country-specific economic status show significant inequity in disease burden attributable to inequity in vaccine uptake. According to the WHO Dashboard (4) presented in Figure 1, LICs and LMICs reported 2 million and 82.8 million confirmed cases, respectively, whereas they reported 430 million and 261 million confirmed cases in HICs and UMICs, respectively, indicating higher reporting in developed nations. In parallel, disparities in vaccine uptake are evident where more than 80% of the population in the HICs and UMICs had at least one dose of COVID-19 vaccine, compared to 69% in LMICs and 36% in LICs (4). Additionally, the economic burden of the pandemic has been very high in LICs and LMICs, with an ∼43% loss in working hours compared to an ∼16% loss in HICs (7). To control this pandemic, the WHO developed the Strategic Preparedness and Response Plan (SPRP), which focuses on an adaptive and agile approach to build and maintain long-term sustainable disease management through an intelligent surveillance system that integrates COVID-19 prevention and control mechanisms in the existing health system (8, 9).

While analysing the situation from a health economic perspective, the need and access gap can be well explained through the concept of healthcare market failure. During the pandemic, market failure has manifested as a multiphase crash in the medical supply chain [in the form of shortages in testing kits, personal protective equipment (PPE), medicines, and vaccines], liquidity crisis—urging for prompt preparedness planning and strategizing to reduce the need-availability gap and increase the resilience in public health system (1, 2, 10–13). Vaccine rollout in 2020 was aimed at limiting the death toll (1.8 million reported deaths with 3 million excess mortalities in 2020) and case fatalities (9). Compared to HICs; LICs and LMICs struggle to access basic health services due to stringency, such as international travel bans, lockdowns, business closures, and employee layoffs, given the context of inequitable social protection mechanism (2, 14). This suboptimal systemic capacity fought further to ensure health market parameters given the greater burden of other communicable diseases, such as HIV/AIDS or tuberculosis (15). Furthermore, infrastructural parameters, such as less availability of hospital beds and limited testing and diagnostic capacity delay the success of pandemic responses in terms of ensuring access to COVID vaccines (15, 16). All these incidents exacerbated the crisis, emphasizing the need for a strategic inclusion of robust innovation-led monitoring framework in pandemic preparedness and response. Participation in global initiatives such as international cooperation and adherence to WHO recommendations (international health regulations) are crucial for LICs and LMICs, but implementation gaps have widened inequities in realizing pandemic response—an outcome of systemic market failure that disproportionately affects poor and marginalized populations (17, 18).

Another contributing factor to healthcare market failure is the commodification of healthcare. It is a historical determinant of healthcare market failure, favouring the affluent section to access expensive skill- and innovation-led services and increasing socioeconomic inequity further (19–22), excluding deprived, vulnerable population subgroups (23–25). For example, a single dose of vaccine was $7 in Uganda compared with $3.50 in Europe, featuring widespread economic disparity due to less effective health protection in lower middle-income countries (5).

In health economics, the concept of health inequity is a central concept applied in policy design to eradicate the socioeconomic gradient of healthcare access and health outcomes in LICs and LMICs. The theoretically well-known measure of health inequity estimates the concentration of a health condition or access to a specific care relative to the socioeconomic status of the population after the population is ranked from the highest level of deprivation to the lowest (23). A large body of research on inequity in health conditions and healthcare access exists; nevertheless, innovative approaches require attention. Among the different innovative technologies under digital health interventions, the application of artificial intelligence (AI) for monitoring health service delivery would support the design of equitable health policy, which is less common in low-resource settings (26, 27). In LICs and LMICs, digital health interventions using AI are mostly at the ideation, design, pilot or feasibility stages with early digital maturity (foundational, developing or scaling) levels compared to the optimized or adaptive phases in HICs (9). Given this context, the current study proposes a novel AI-driven framework for monitoring global vaccination inequity. This study explored the macrolevel determinants of COVID-19 vaccination inequity at the global scale by applying classical and machine learning algorithms to an integrated dataset created from global data sources for a select period of 3 years.

## Determinants: a pathway towards the theoretical frameworks

Different studies have explored the demand-side correlates of vaccine uptake, including social (gender, education, ethnicity), behavioural (attitude), perceptual (efficacy, side effects, cost), and accessibility factors (28–30). On the supply side, exploring the availability dimension of access reveals that prioritization-based resource allocation following the standard implementation framework is crucial to correct market failure in vaccine access (2, 5). The current study explores the determinants of vaccination inequity from a macroeconomic perspective; built on national-level indicators, it compares the regional variation in vaccination inequity to feed global health equity policy.

Research over the past 5 years has explored the factors associated with widening accessibility gaps during the COVID-19 pandemic. Studies have shown that service providers face challenges in delivering routine public health services, especially maternal and child health (MCH) services, due to a lack of capacity and resource shortages (31, 32). Ashish et al. (32) reported that COVID-19 stringency measures resulted in falls in antenatal checkups and facility births in indigenous communities. As evident from Riley et al. (33), a 10% decline in MCH service uptake is evident, which threatens the decadal effort of universal health coverage. Another study exploring the impact on services targeting noncommunicable diseases revealed that the utilization of existing limited resources for pandemic response affected access to routine treatment of chronic ailments; patients with diabetes were the most impacted (38%), followed by those with COPD (9%), hypertension (8%), heart disease (7%), asthma (7%), cancer (6%), and depression (6%) (34). In addition, traditional health monitoring systems are unable to track and manage COVID-19 testing gaps and patient flow, thereby further straining scarce resources (35) requiring a shift in monitoring production frontier during and after disaster (36). Additionally, the lack of efficient reporting of migration patterns has complicated containment efforts in high-risk countries (37–39). Furthermore, with gaps in sectoral coordination, preparedness planning faces challenges in ensuring testing and quarantine services (37, 38, 40).

Inequity in access to vaccines remains a persistent global health challenge, especially in LICs and LMICs, where suboptimal structural and systemic readiness hinder equitable access to immunization and make the vaccination progress inconsistent (41). Evidence from WHO African region depicts the influence of structural and several institutional factors on vaccine uptake (42, 43). Likewise, evidence from Palestine emphasizes the role of inter-group dynamics (Bedouins—non- Bedouins) negatively influence vaccine uptake among minor groups (41, 44). Alike, marginalized communities in Slovakia (Roma communities) faced significant difficulties in terms of disparate coverage by insurance companies, shortage of health worker, ill-treatment of health workers while accessing different vaccination services (41, 45). Another study has shown that Community-centred service lines having localized arrangements can address the access related disparities and increase equal coverage (46). Broader reflections from the editorial views of Amponsah-Dacosta et al. (41) on immunization services during coronavirus pandemic underline how COVID-19 disruptions impacted vaccination services thereby widening pre-existing inequities in access to vaccines globally.

**Figure 2:**
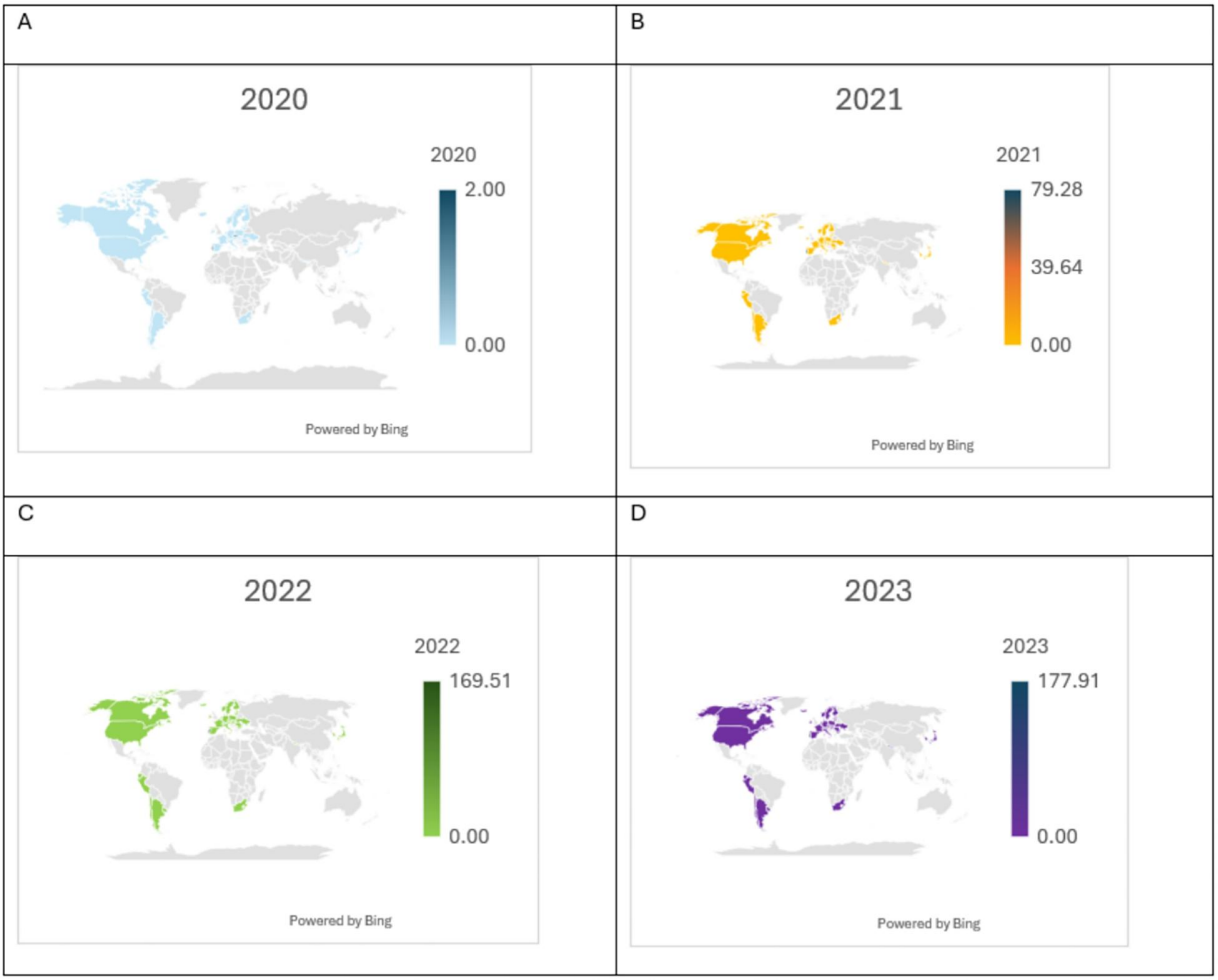
Average of COVID-19 doses (cumulative) from 2020 to 2023: manufacturer Covaxin - **(A)** 2020, **(B)** 2021, **(C)** 2022, and **(D)** 2023.

Given such an impact on a less resilient system, mass vaccination against COVID-19 has become a mandate for overcoming the spread of the pandemic. The WHO, GAVI, the vaccine alliance, and the Coalition for Epidemic Preparedness Innovation (CEPI) co-led COVAX—manufacturing and rollout from early 2020. Although several LMICs have well-built immunization program management systems, more than half of developing countries lack efficient programs—operational and technical support for vaccination (47, 48). COVAX was aimed at a population-proportional model; however, it was found to be less compatible with fairness-based prioritization—vaccine distribution experienced restructuring in supply and dose administration to increase its global coverage. Figure 2 exhibits average Covaxin dose in 2020 (A), 2021 (B), 2022 (C), 2023 (D). One of the major tasks has become crucial to follow the WHO monitoring framework, side by side, identifying barriers to accessing vaccination in LICs and LMICs.

Research investigating the barriers to access immunization revealed the influence of social determinants in low-resource settings (27, 49). Most of these inferences rely on traditional regression models (50). The potential of digital health using artificial intelligence (AI) is an emerging field of interdisciplinary research that explores the applicability of innovative machine learning (ML) algorithms and finds that the ML model outperforms the classical model. These studies demonstrate the ability of advanced analytics to identify determinants of healthcare access with higher accuracy and precision and increasing the predictability power of the models (51–55); nevertheless, lack of research on inequality in access to vaccine is evident when focusing on global policies for low-resource settings (27).

In the last 5 years, many studies have explored the applicability of ML models in identifying the correlates of immunization. Some of them focused on vaccination inequity, and some concentrated on COVID-19 vaccination inequity. Among those studies, one explored the factors responsible for zero-dose children in Tanzania via logistic regression (LR), decision tree classifiers (DT), random forest classifiers (RF), support vector machines (SVM), K-nearest neighbors (k-NN), eXtreme gradient boosting (XGBoost/XGB) and naïve Bayes (NB) (56). RF was found to be the best model, with an accuracy of 0.95, precision of 0.94, recall of 0.96, *F*1 score of 0.95 and AUROC of 0.99 (56). A study predicting incomplete immunization among children under 5 years of age revealed that the XGBoost classifier was the best performer, with an accuracy of 0.79, a recall of 0.90, an *F*1 score of 0.81, a precision of 0.74, and an AUROC of 0.86 (57). They also identified the important features using Shapely additive eXplanation after running XGBoost models (57). Another study by Rustagi et al. (58) explored the effectiveness of COVID-19 vaccination using ML algorithms, and SVM was used to analyse the efficacy of the linear and polynomial models generated. While exploring the structural determinants of vaccination inequity, ML models are used to evaluate the effectiveness of stringency regulations in terms of the associations between lockdown protocols and infection rates (59), and flexible models are used to determine the associations between lockdown procedures and disease transmission patterns (60). A study by Shayegh et al. (61) proposed an AI-based novel framework for prioritizing COVID-19 vaccine allocation in resource-scarce settings such as Kenya. The authors used vulnerability features to gather country-specific data on demographic, socioeconomic, epidemiological, healthcare, and environmental factors. Then, the prioritization of vaccination is estimated by calculating a vulnerability Index using a set of covariates weighted using AI. Finally, values are considered by identifying the most effective GIS-assisted context-specific constraint adjusted vaccine allocation at the local scale. According to this study, only 45% of socially vulnerable groups are vaccinated, and coverage significantly increases if a new framework is adopted. Kazemi et al. (27) estimated the vaccination uptake rate using a least square fitting algorithm to fit the rate of change in cumulative cases of a logistic growth model followed by estimation of the vaccine rollout index using uptake rate, the total number of doses administered and the total population. They found the significance of socioeconomic disparity in influencing vaccine uptake and used RF algorithm to predict important influencing features. They reported that the median per capita income and human development index are the top influencing features, indicating inequity in vaccine uptake by economic factors (27). Kaur et al. (62) proposed the VaxEquity framework for COVAX countries using ML models for risk assessment and optimization. ML algorithms identify determinants of vaccination, and an optimization framework is developed to enhance the equitable distribution of vaccination. Nair (63) applied integer linear programming and heuristic methods to develop an AI-driven optimizer. The novel ADVISER developed by these authors outperformed baseline interventions for increasing vaccine uptake through experimental evaluation. Carrieri et al. (64) applied an ML model to area-level data to predict vaccine hesitancy, and the ML models performed effectively in making predictions with socioeconomic and demographic features.

Given this background, this research is an attempt to reflect a few indicative directions to correct market failure, proposing AI-based framework testing with secondary data sources. Barriers to accessing vaccines are used to design a framework based on the above background research and are tested to understand the market failure-related correlates in aggravating COVID-19 vaccination equity. This knowledge is generated through robust predictive analysis to provide digital solutions to accelerate data-driven decision making and optimize resource allocation toward future pandemic preparedness and response.

Based on the abovementioned review of the problem, the research questions addressed by the current study are as follows:

### Research questions

1. What are the region-specific differences in vaccination rates and region-specific vaccination inequity?
2. What are the structural determinants at the macro level that determine spatial vaccination inequity?
3. Does machine learning perform better than classical binary logistic regression in identifying the correlates of vaccination inequity for equitable policy design?

### The theoretical framework

**Figure 3:**
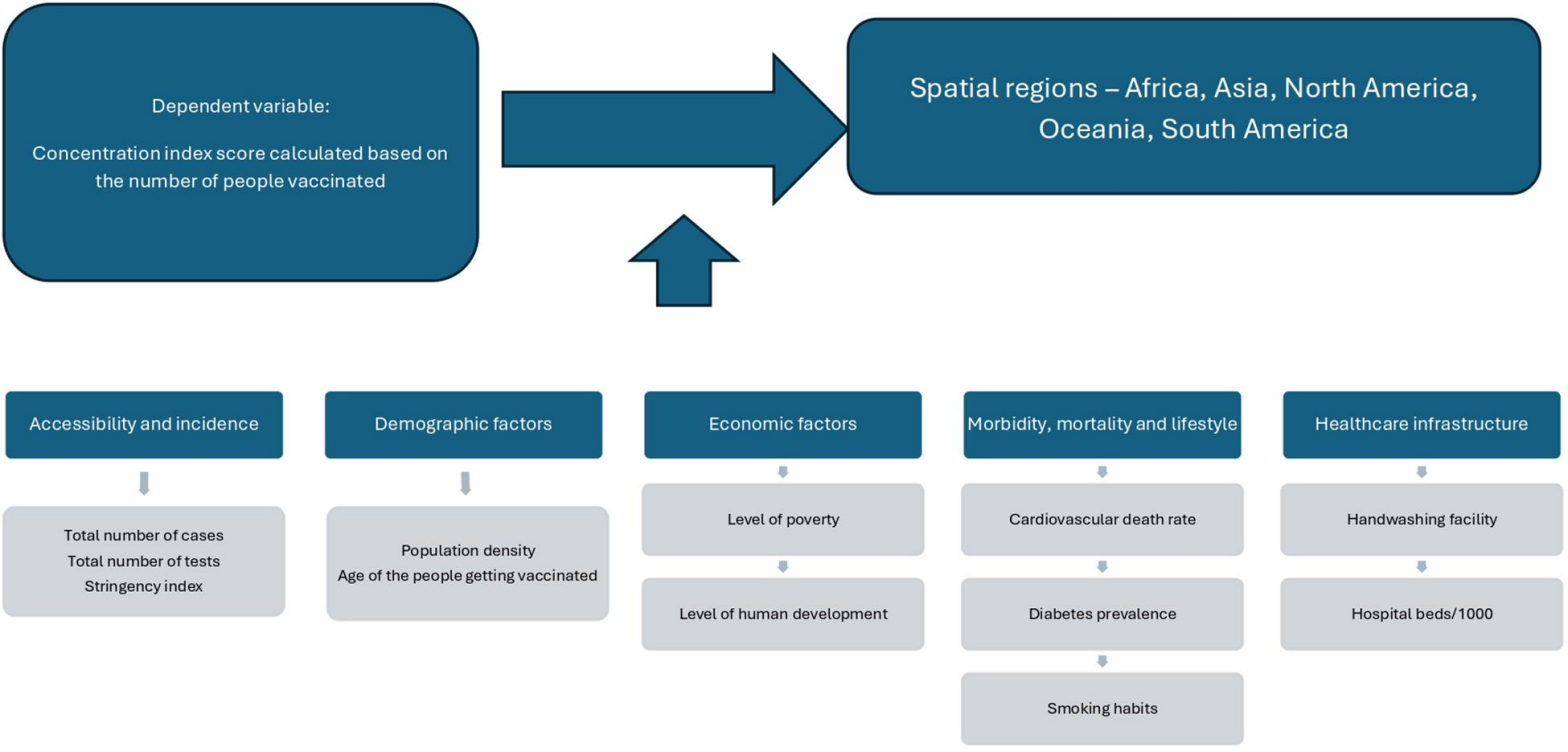
The theoretical framework.

Therefore, the theoretical framework of the determinants of spatial vaccination inequity demonstrated in Figure 3 is as follows:

The theoretical framework consists of a dependent variable, an independent variable and controls. The variables are explained in Table 1.

**Table 1:** Variable description.

Variable	Type	Description
Dependent variable: binary form of Concentration index score	Binary	Ranking the countries by the socioeconomic status measured by gross domestic product (GDP) from poorest to richest and estimating the cumulative distribution of getting vaccinated based on GDP distribution
Observational unit –		
Countries covering vaccination uptake per day from 2020 to 2023		
Independent variable—Spatial regions—the continents	Nominal, categorical	Africa
		Asia
		North America
		Europe
		Oceania
		South America
Controls		
Accessibility	Continuous	Total number of tests performed
Incidence	Continuous	Total number of cases
Stringency index	Continuous	A composite index based on the measures under COVID 19, for example, staying at home, no travel, school closure, no gathering
Population density	Continuous	Population per unit of the area
Age	Continuous	Median age of the population
Age 65 or older	Continuous	% of population in the respective age group
Age 70 or older	Continuous	% of population in the respective age group
Extreme poverty	Continuous	% of people below poverty line
Human development index	Continuous	Score of human development comprising literacy rate, life expectancy and per capita income
Cardiovascular death rate	Continuous	Number of deaths from cardiovascular disorder per 1,000
Diabetes prevalence rate	Continuous	% of people diagnosed with diabetes
Female smokers	Continuous	% of female smoke any kind of substance
Male smokers	Continuous	% of male smoke any kind of substance
Handwashing facilities	Continuous	% of facilities available
Hospital beds per thousand	Continuous	Number of hospital beds available per 1,000 population
Year	Continuous	From 2020 to 2023

To address the research questions, the following hypotheses are proposed:

- H1: The concentration of pro-poor or pro-rich vaccination varies by continent.
- H2: Continent-level heterogeneity in the concentration of getting vaccinated is influenced by the abovementioned five sets of factors.
- H3: The explainability of machine learning models is greater than those of classical regression models.

## Methods

### Data source

**Figure 4:**
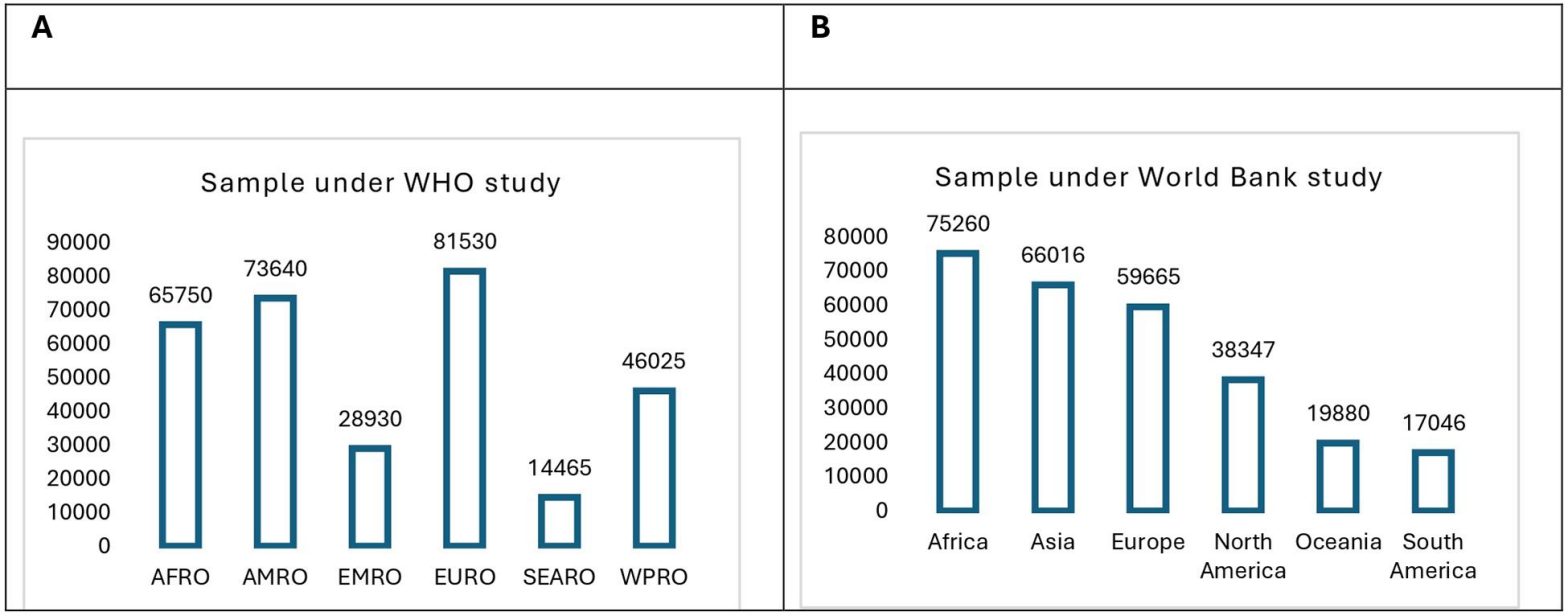
**(A)** Sample under the world health organization (WHO) study and **(B)** sample under the world bank (WB) study.

The analyses were conducted using two secondary datasets—the World Health Organization COVID-19 Dashboard (2023) and the World Bank COVID-19 dataset (2023). Sample under WHO and WB datasets are presented in Figure 4. The WHO study reported vaccination data from 107 countries from WHO regions as observational units—Africa (AFRO), Americas (AMRO), Eastern Mediterranean (EMRO), Europe (EURO), Southeast Asia (SEARO), Western Pacific (WPRO). The World Bank survey collected information on immunization/vaccination uptake per day from 195 countries (1,420 data points/country), covering 276,900 observations (from 07.12.2020 to 27.11.2023) from WB regions classified as High-Income Countries (HIC), Upper-Middle-Income Countries (UMIC), Lower-Middle-Income Countries (LMIC), and Low-Income Countries (LIC). The final dataset contains 60,339 observations after deleting the days where the total number of vaccinations reported is “0”. Additionally, country-specific demographic factors, economic factors, morbidity, mortality rates, and healthcare infrastructure availability are pooled from different secondary sources in count, mean/median or percentage form according to the definitions of the indicators, which are available in Our World in Data (65).

### Data analysis

Both exploratory and predictive analyses were conducted. Region-specific analyses are conducted where regions are classified according to the WHO classification of regions (the Americas, European and Eastern Mediterranean countries, African continent, Southeast Asia, and Western Pacific) and the World Bank classification of country groups (high-income, upper- and middle-income, lower-middle-income, and low-income). The standard concentration indices are estimated following the definition in Wagstaff et al. (23) by region (continents) to explore the degree of inequity by region. To estimate the odds of vaccination inequity by spatial region, classical binary logistic regression models are run. The case incidence, sociodemographic factors, prevalence of morbidity, mortality, lifestyle behaviour, and institutional capacity of the healthcare system are adjusted to explore the nonlinearities in the spatial variation in vaccination inequity with phased incorporation of factors. The estimation of the concentration score is followed by 9-phase multivariate analysis. Year is incorporated in Model 9 to capture the influence of time on vaccination inequity across regions.

Under classical regression analysis, 9 binary logistic regression models are run where Model 1 estimates the associations between geographical regions and the occurrence of pro-poor or pro-rich vaccination inequity. Gradually, different factors from the theoretical framework are inserted to estimate the adjusted odds of region-specific vaccination inequity. Next, 7 machine learning models—logistic regression (LR)—the base model, decision tree (DT), random forest (RF), XGBoost (XGB), naïve Bayes (NB), light gradient boosting machine (LightGBM), and extremely randomized trees (extra trees)—are run to find the best prediction model measured by different evaluation metrics.

### Model evaluation and weighted scoring approach

To identify the most appropriate supervised learning classifier for predicting COVID-19 vaccination inequity between 2020 and 2023, ML models are evaluated using three key metrics: accuracy, *F*1 score (balancing precision and recall), and area under the receiver operating characteristic curve (AUROC). Recognizing the importance of balancing type I and type II errors in identifying the correlates of vaccination inequity, a weighted scoring system is employed to rank the models by performance. Weight assignment followed ML literature in public health (66). The current study applied equal weight to the metrics Accuracy, *F*1 score, and AUROC to avoid any ambiguity. The deduction of differences in weight values among the metrics is beyond the scope of this research. Precision and Recall are not included to avoid double counting as *F*1 score is considered.

The final weighted score for each model was computed as:

Score=0.33×accuracy+0.33×F1+0.33×AUROC

This composite scoring allowed for robust and equity-sensitive comparisons—well considering all the metrics with equal importance. The best model is identified after the models are ranked based on the weighted score estimated as the additive value of all the metric scores multiplied by their assigned weights. In addition to this further, the best model selection criteria also includes whether the model is free from overfitting. Considering this point, 5-fold cross-validation is applied on each model, estimated the standard deviations to assess the variability across the folds. The model with high AUROC with less overfitting is selected. StratifiedGroupKFold is used for cross-validation. For each model a pipeline is created encapsulating SimpleImputer, SMOTE, StandardScalar (where applicable) before the model itself. SMOTE is applied to the training folds. This ensures that preprocessing steps are applied within each fold, preventing data leakage. The “Year” variable is used as the grouping variable for the cross-validation representing temporal influence.

## Results

### The world status (during the period of the study)

**Figure 5:**
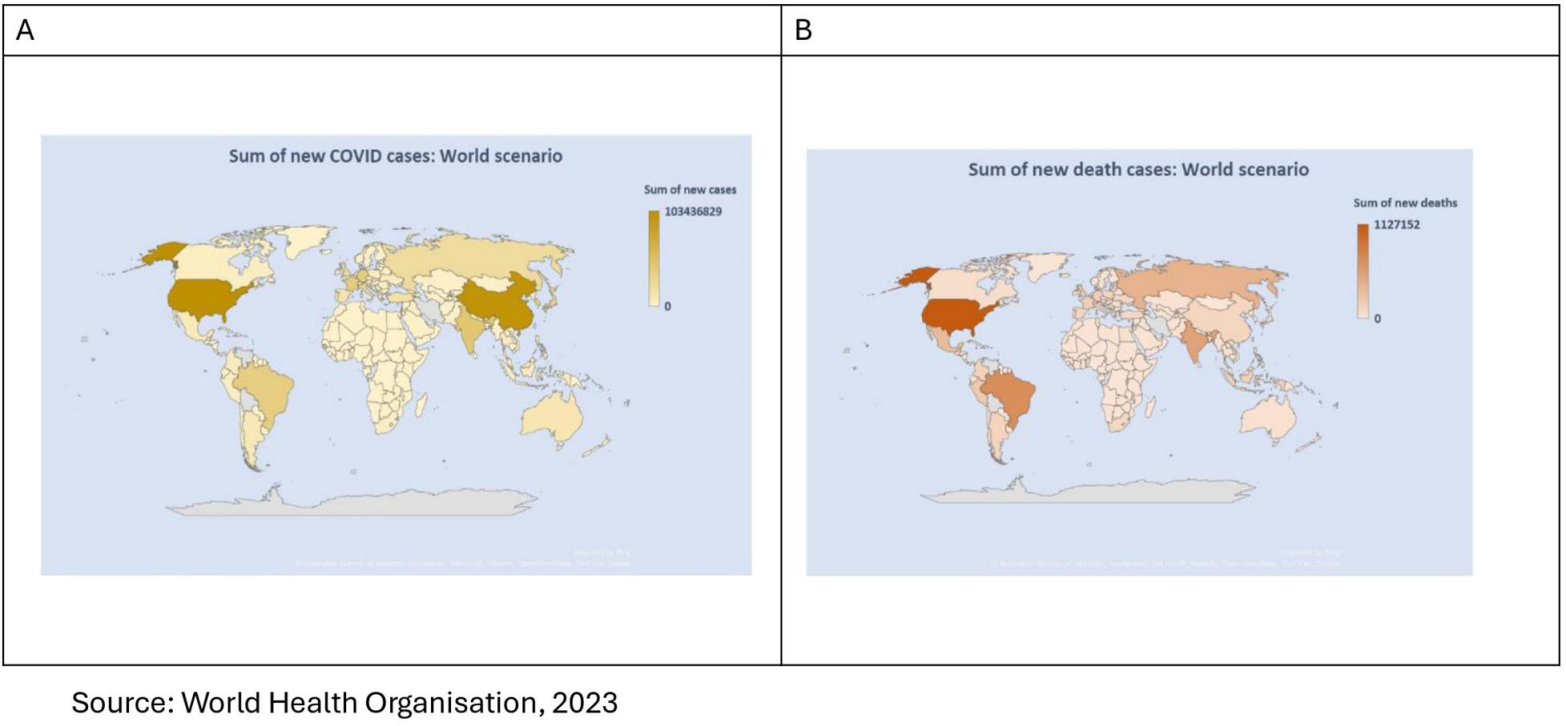
**(A)** Sum of new COVID-19 cases and **(B)** Sum of new death cases attributable to COVID-19—the world scenario.

**Figure 6:**
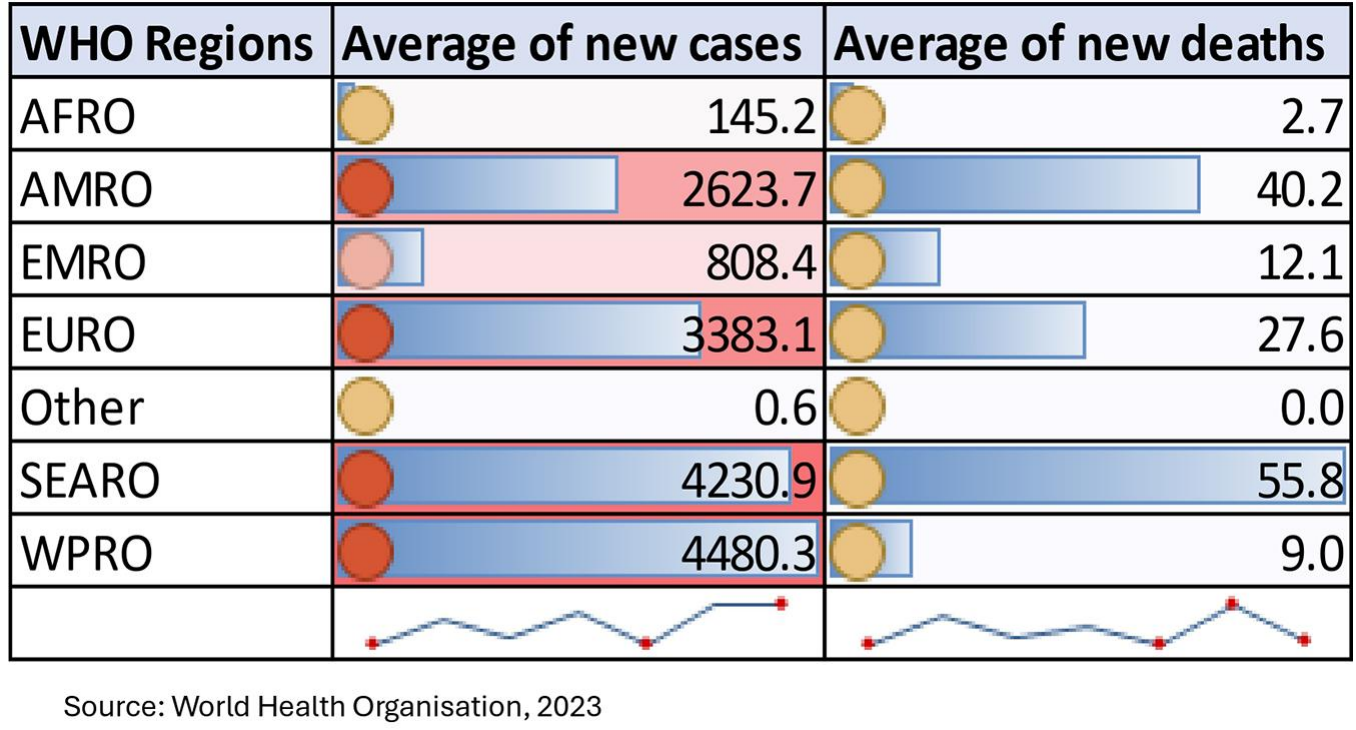
Average number of new cases and deaths by WHO classification of countries.

Figure 5 clearly shows that the second phase of the pandemic was highly concentrated in the Americas and, to some extent, in Asia, and the African continent. Among low- and middle-income countries, the reported death toll is higher in the eastern and southern parts of Africa, East Asia and Southeast Asia. It is also evident from Figure 6 that the average number of new cases is greater in EURO, SEARO and WPRO, whereas the average number of deaths is greater in the SEARO region. Therefore, this may be one of the reasons why accessibility issues are retarding universal coverage of vaccination, resulting in inequity in health status.

**Figure 7:**
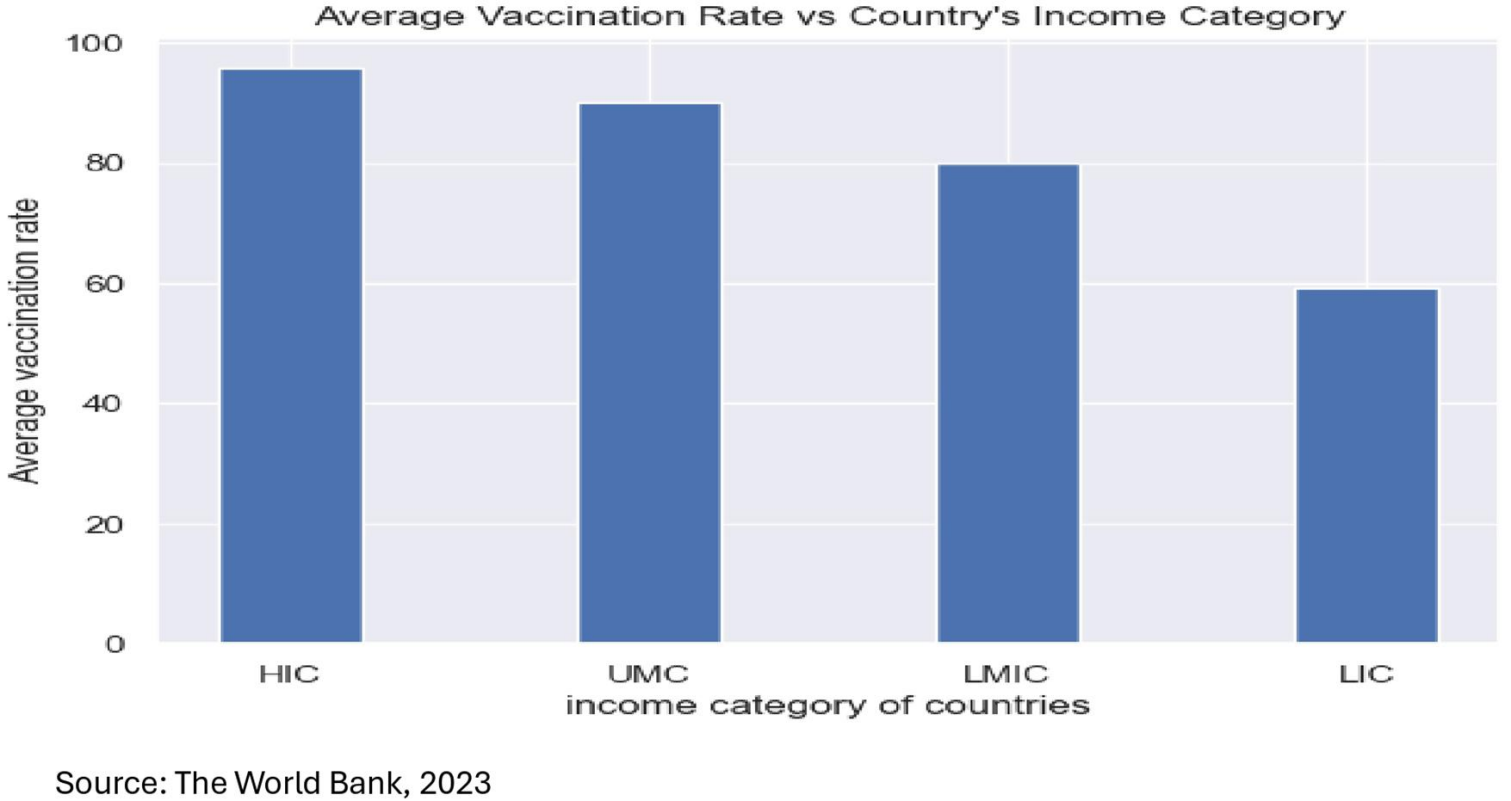
Average vaccination rate by the world bank classification of countries.

Figure 7 shows that a country's GDP is positively associated with average vaccination rates. This finding indicates that lower vaccine uptake rates, and suboptimal access to healthcare infrastructure, less access to vaccines, lower public health effectiveness, less availability of pandemic control initiatives, lack of good stringency are associated with varying degrees of vaccine uptake in low-income countries and lower- middle-income countries.

**Figure 8:**
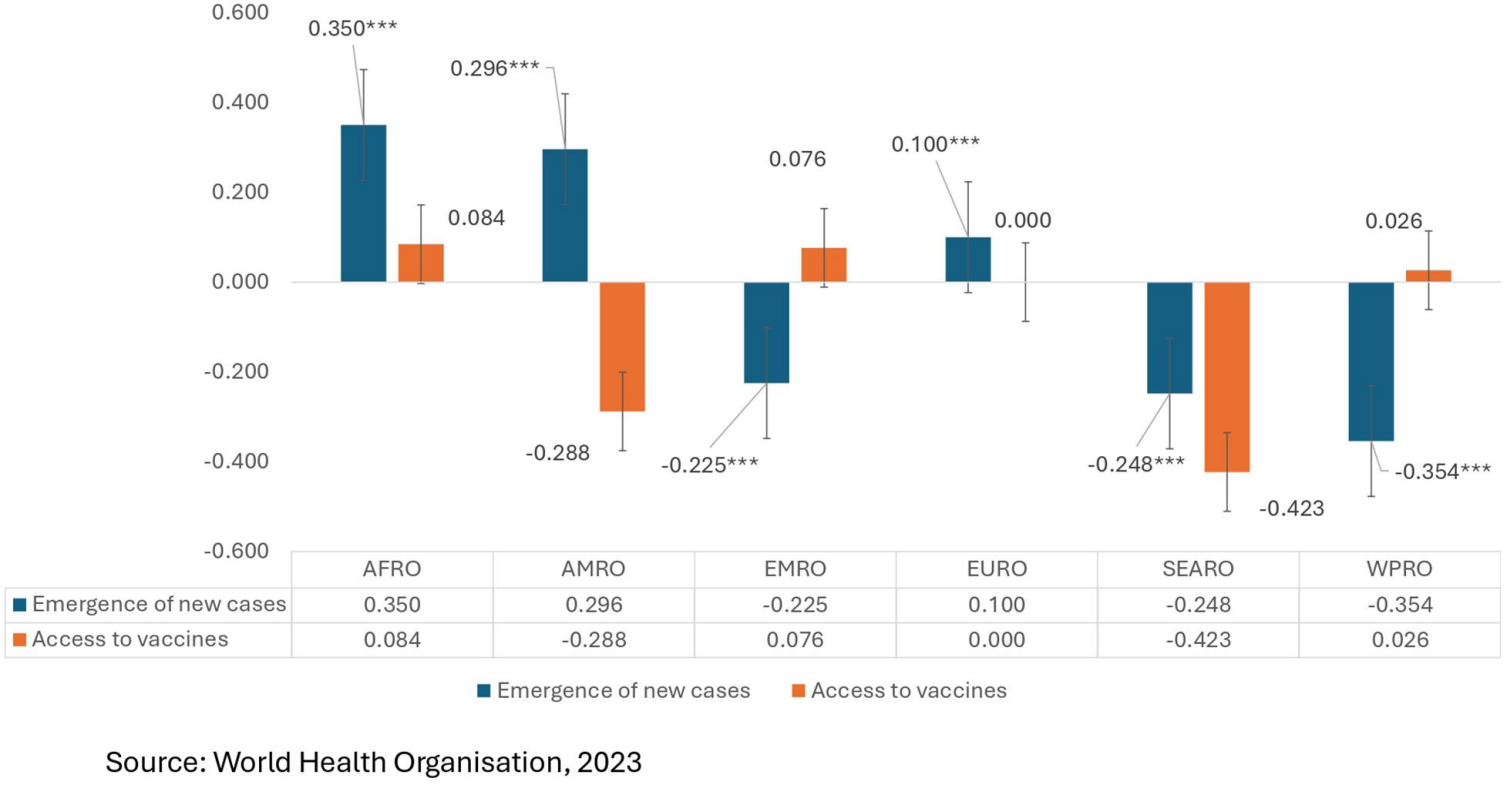
Regional inequity (CI) in the new cases and vaccine uptake ranked by countries in a region.

To investigate regional inequities according to the WHO classification of regions, Figure 8 shows that inequity in the pattern of new cases was highly concentrated among richer countries in the AFRO and AMRO regions at the 99% level of significance. The EURO region shows that a CI value of +1 may imply a higher concentration of reporting in richer countries, and under- or no reporting is visible in less resourced countries. The CI values of new cases are highly concentrated among the low-resource settings in EMRO, SEARO and WPRO at the 99% level of significance. The comparatively higher incidence in richer countries within the African subcontinent might be due to underreporting in poorer settings or greater cross-border travel among richer populations, contributing to the emergence of new cases. Although the American region depicts higher incidence rates within high-income settings, this may be attributed to spread of infection facilitated by international travel. The concentration index values with respect to vaccine access are significant only in the EURO region, reflecting equitable access across both poorer-resource and richer-resource settings.

**Figure 9:**
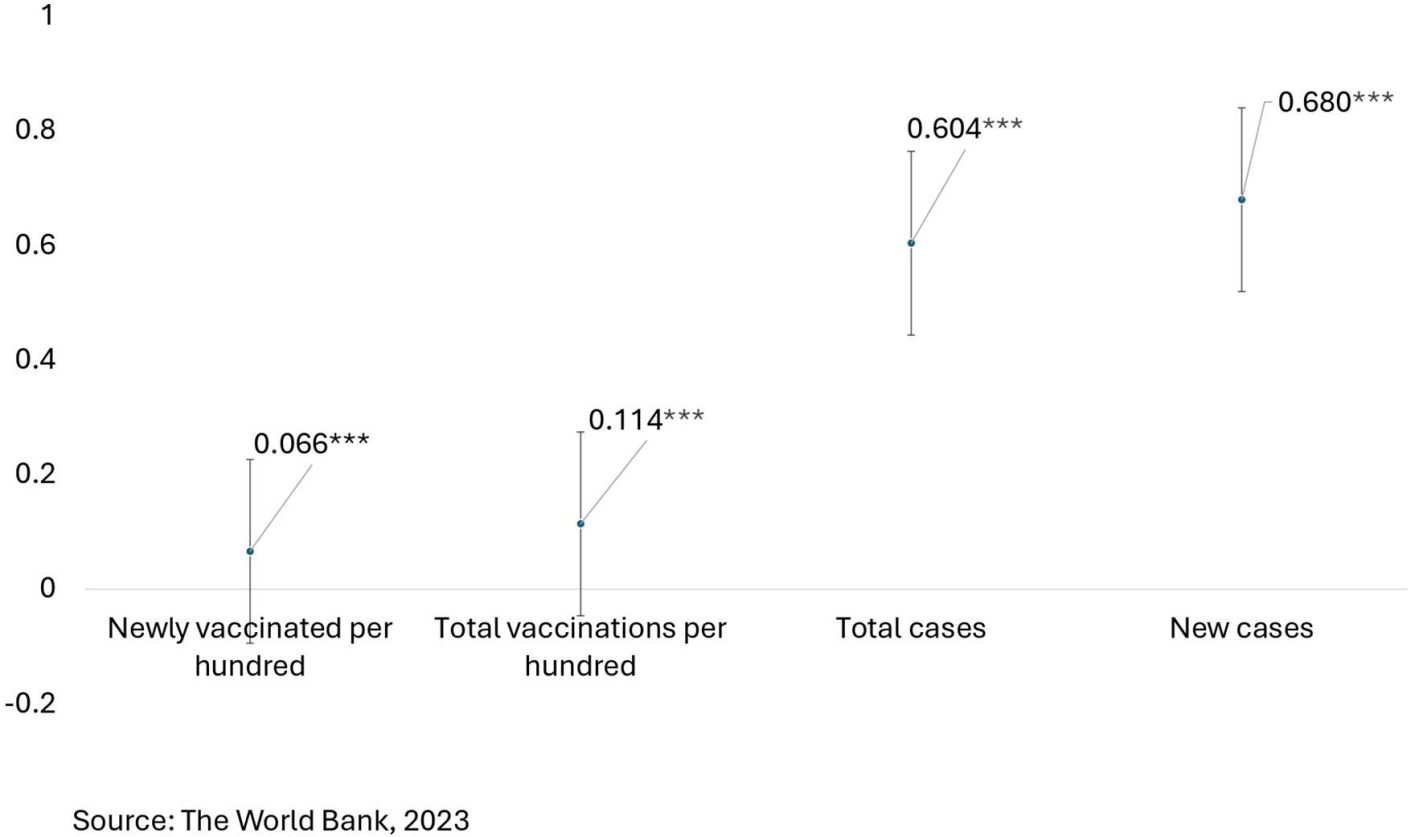
Four types of vaccination inequity -concentration indices for four different phenomena.

Figure 9 exhibits how socioeconomic inequality is associated with four COVID-19 outcomes measured using concentration indices. Newly vaccinated per 100 reflects slight pro-rich inequity (0.066*), indicating relatively higher vaccination coverage among wealthier countries at the time of data collection. The total number of vaccinations per 100 is pro-rich (0.114*), reflecting that total vaccination coverage is also greater among wealthier populations. Total cases (0.604*) and new cases (0.680*) are strongly pro-rich, reflecting that high number of reported cases in wealthier countries, which may be due to higher testing capacity, greater international mobility and more urbanized living practices.

**Figure 10:**
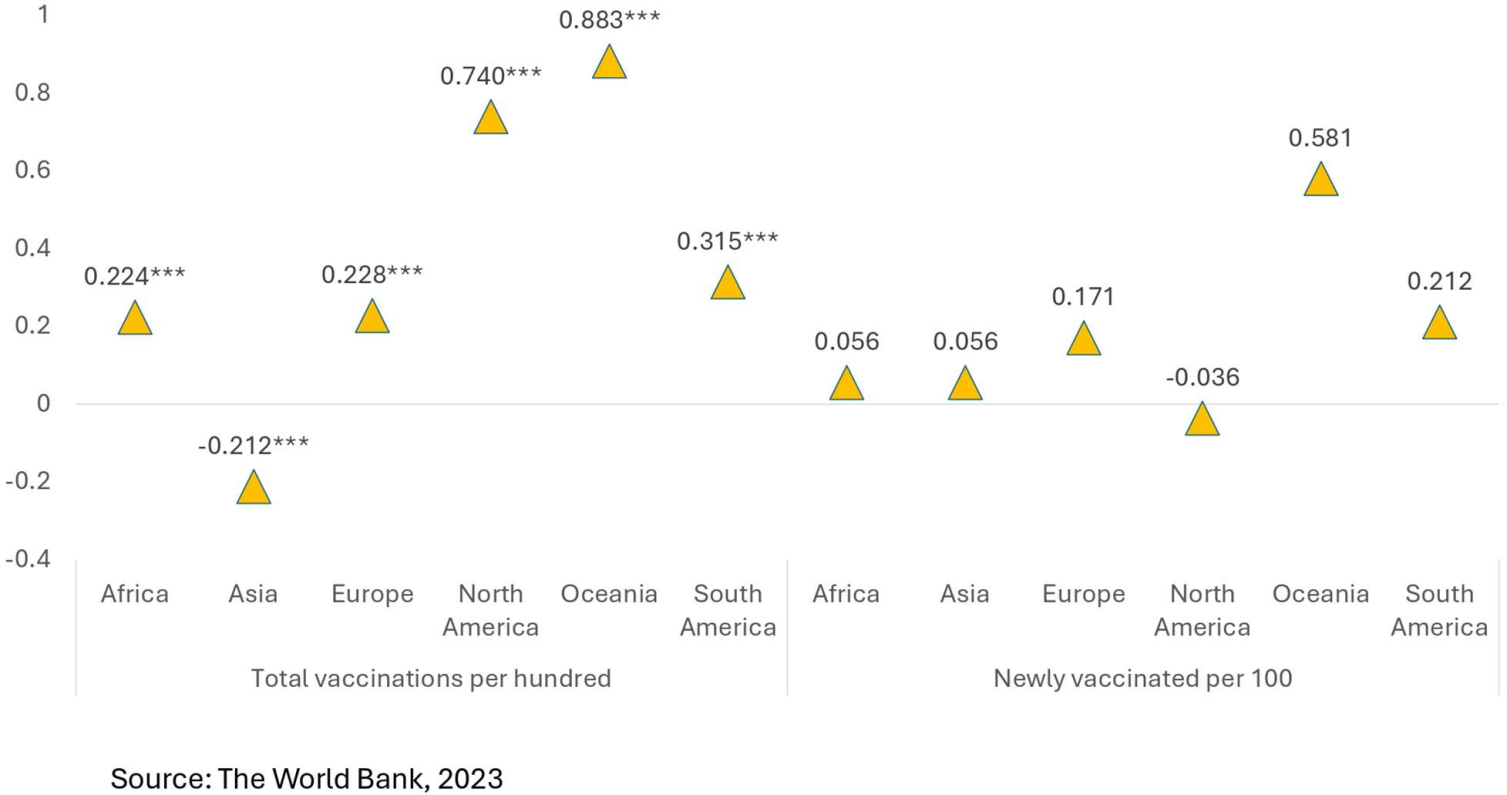
Inequity in total vaccinations per 100 and inequity in newly vaccinated per 100 by spatial location.

Figure 10 shows inequity in total vaccinations per 100 people and inequity in newly vaccinated individuals per 100 people by spatial location during the time of the study.

The total number of vaccinations per 100—African continent reflects pro-rich (0.224***) inequity, indicating that richer African nations have more vaccinations than poorer ones do. It is more pro-poor (−0.212***) in Asia; in other words, low-resource settings had more access to vaccines. Wealthier European countries are better vaccinated (0.228***). North America and Oceania show strong pro-rich inequality (0.740*** and 0.883***, respectively), and South America shows a moderate pro-rich pattern (0.315***).

Newly vaccinated per 100—There is slight pro-rich inequality in relation to new vaccinated cases in Africa (0.056***). Mild inequality favouring richer populations is evident in Asia (0.056***). Vaccine rollout is moderately pro-rich in Europe (0.171***) and South America (0.212***), highly pro-rich in Oceania (0.581***), and a slight pro-poor shift is evident in North America (−0.036**).

Therefore, in summary, it can be inferred that vaccination is pro-rich globally, especially in Oceania, Europe, and North America. Asia depicts pro-poor inequality, possibly due to mass vaccination initiatives by health programmes in LMICs supported by global initiatives like COVAX. North America has experienced a slight shift from pro-rich to pro-poor and may have targeted efforts to increase coverage among underserved populations. Regional vaccination efforts are inequitable, with some shifts toward a pro-poor distribution. With that said, wealth and prosperity are still significant predictors of access. Therefore, vaccine coverage strategies would be more equitable with targeted outreach in high inequity regions.

Table 2 presents the results of classical logistic regression models. The models show regional comparisons relative to Europe, North America and Oceania (HICs or richer countries) and the three other regions—Africa, Asia, and South America. The main model shows that, relative to richer countries, the likelihood of pro-poorness of the vaccine access is lower in other continents (in Africa, Asia, and South America). In other words, access to vaccines in low-resource settings in Africa (OR = 0.027; 99%), Asia (OR = 0.213; 99%), and South America (OR = 0.191; 99%) is evidently lower than in richer continents.

**Table 2:** Logistic regression models exploring the determinants of vaccination inequity.

	Main Model	Model 2	Model 3	Model 4	Model 5	Model 6	Model 7	Model 8	Model 9
Dependent variable: Binary variable created from concentration score <0: pro-poor (value = 1); >0: pro-rich (value = 0)	Continents as main independent variable: base (Europe, America, Oceania)	+No. of cases and tests	+Degree of stringency	+Demographic factors	+Economic factors	+Burden of non-communicable disease	+Lifestyle factor	+Service availability and appropriateness	+Year
Africa	0.027***	0.029***	0.030***	0.408***	0.733***	1.908***	3.774***	8.821***	8.853***
Asia	0.213***	0.222***	0.238***	0.593***	0.327***	1.186***	1.743***	2.601***	2.948***
South America	0.191***	0.179***	0.186***	0.545***	0.727***	0.236***	0.256***	0.349***	0.355***
Total cases		1.043***	1.031***	0.995	1.024***	1.085***	1.044***	1.030***	1.060***
Total tests		1.011***	1.022***	1.014***	1.010***	0.984***	0.979***	0.984***	0.974***
Stringency index			0.992***	0.989***	0.990***	0.994***	0.999	0.997***	0.990***
Population density				0.976**	1.275***	0.780***	0.995	1.281***	1.267***
Median age				1.234***	1.141***	1.134***	1.264***	1.301***	1.299***
Aged 65 or older				1.360***	1.502***	1.272***	0.796***	0.733***	0.795***
Aged 70 or older				0.665***	0.559***	0.872***	1.004	0.839***	0.759***
Extreme poverty					0.470***	0.514***	0.472***	0.540***	0.542***
Human development index					9.925***	1.300	0.276***	0.179***	0.193***
Cardiovascular death rate						0.988***	0.987***	0.981***	0.981***
Diabetes prevalence						1.174***	1.186***	1.312***	1.307***
Female smokers							1.320***	1.441***	1.442***
Male smokers							0.976***	0.964***	0.964***
Handwashing facilities available								1.010***	1.009***
Hospital beds per thousand								2.088***	2.055***
LR Chi^2^	14,116.480	14,408.579	14,726.196	31,922.765	36,860.839	43,559.465	48,705.961	51,206.475	51,363.263
*p* value	0.0000	0.0000	0.0000	0.0000	0.0000	0.0000	0.0000	0.0000	0.0000
*N*	60,339	60,339	60,339	60,339	60,339	60,339	60,339	60,339	60,339

* *p* < 0.10.

** *p* < 0.05.

*** *p* < 0.01.

To explore the differences in inferences from bivariate and multivariate analyses, different factors are controlled to check whether they play any confounding role. After adjusting for the number of cases and tests, the pro-richness remains. Stringent policies have a minor but significant linkage. After adjusting for demographic and economic factors, the direction of inequity in vaccine uptake did not change. LICs and LMICs in Africa (aOR = 1.908; 99%) and Asia (aOR = 1.186; 99%), with higher burdens of noncommunicable diseases, are more likely to experience pro-poor access to COVID vaccination. While controlling for the burden of noncommunicable diseases, people suffering from chronic ailments are vaccinated on a priority basis, as indicated by the models that countries with higher NCD burdens may have prioritized these vulnerable groups more equitably. After controlling for behavioural factors, it is evident that pro-poor access in LICs and LMICs in Africa (aOR = 3.774; 99%) increased, whereas in Asia (aOR = 1.743; 99%), pro-poorness in vaccine access remained comparatively high but lower compared to the African region. Thus, it can be inferred that a higher concentration of vaccine uptake favouring poor individuals in LICs and LMICs in African and Asian continents increases where structured public health policies target people with higher burden of noncommunicable disease or worse lifestyle behavior, implying that these individuals receive vaccines if they are exposed more to health risks. The odds of favouring poor individuals increase significantly with the availability of basic healthcare services such as hygiene facilities or hospital beds—Africa (aOR = 8.821; 99%) and Asia (aOR = 2.601; 99%). The pro-poorness characteristics increases further in LICs and LMICs in Africa (aOR = 8.853; 99%) and Asia (aOR = 2.948; 99%) after controlling the temporal factor in Model 9. With respect to model fitness, the LR Chi^2^ increases from 14,116.480 to 51,363.263, with a *p* value <0.001, reflecting that structural factors over time explain a significant narrowing of spatial inequity favouring the LICs and LMICs in African and Asian regions.

### Machine learning models

**Figure 11:**
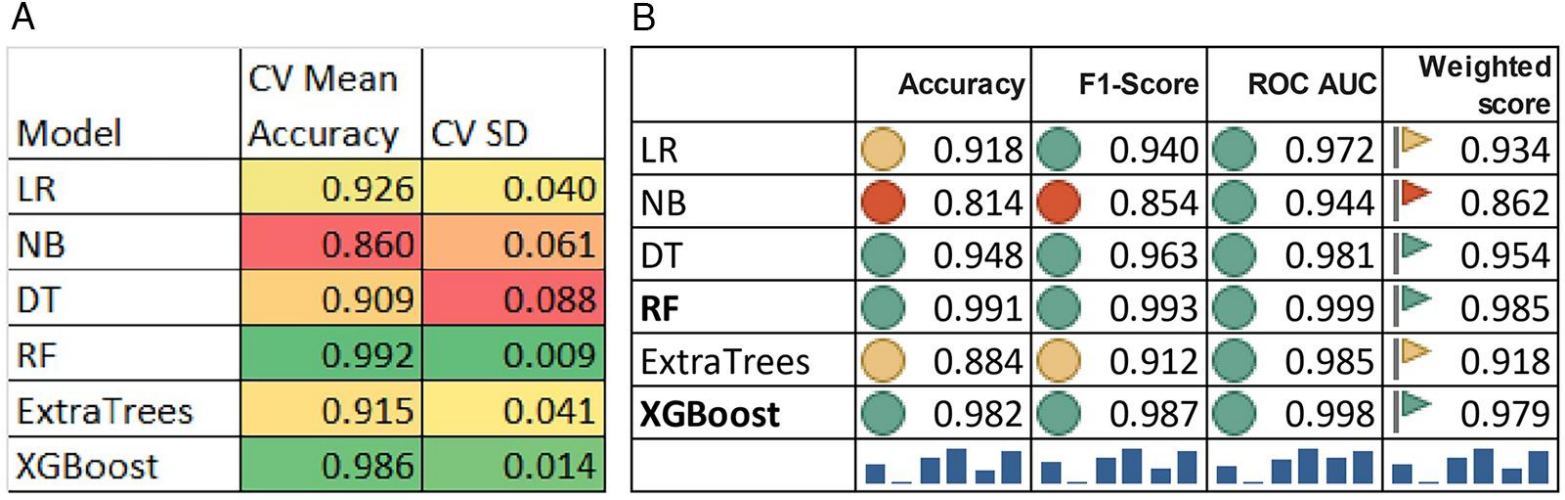
**(A)** Five-fold cross validation with temporally stratified splits. **(B)** Performance comparison of ML Models—LR, DT, RF, XGB, NB, and extra trees.

To run ML algorithms, the dataset is divided into a training set (80%) and a test set (20%). Synthetic Minority Over-sampling Technique (SMOTE) is run after Train-Test split to balance the data imbalance. A 5-fold cross-validation technique stratified using time variable “year” is used to efficiently use the train–test splits to increase generalizability, prevent overfitting and obtain stable estimates while reducing the variance may occur due to the random split of data into training and test sets. For the 6 machine learning models, across the five cross-validation splits, the models demonstrated stable performance. The outcome variable categories are “Pro-Rich uptake” and “Pro-Poor uptake” which was moderately imbalanced before SMOTE. Before SMOTE, number of observations in “Pro-Rich uptake” was 14,279 in the train set which is balanced after SMOTE and now contains 33,992 observations which is equal to the number of observations in the class “Pro-Poor uptake” in the train set. The low standard deviation in performance metrics (approximately 0.009 for Random Forest, 0.014 for XGBoost) suggests minimal variability across data splits, indicating consistent model behaviour and reliable performance estimation (Figure 11A).

### Performance evaluation of the ML models

**Figure 12:**
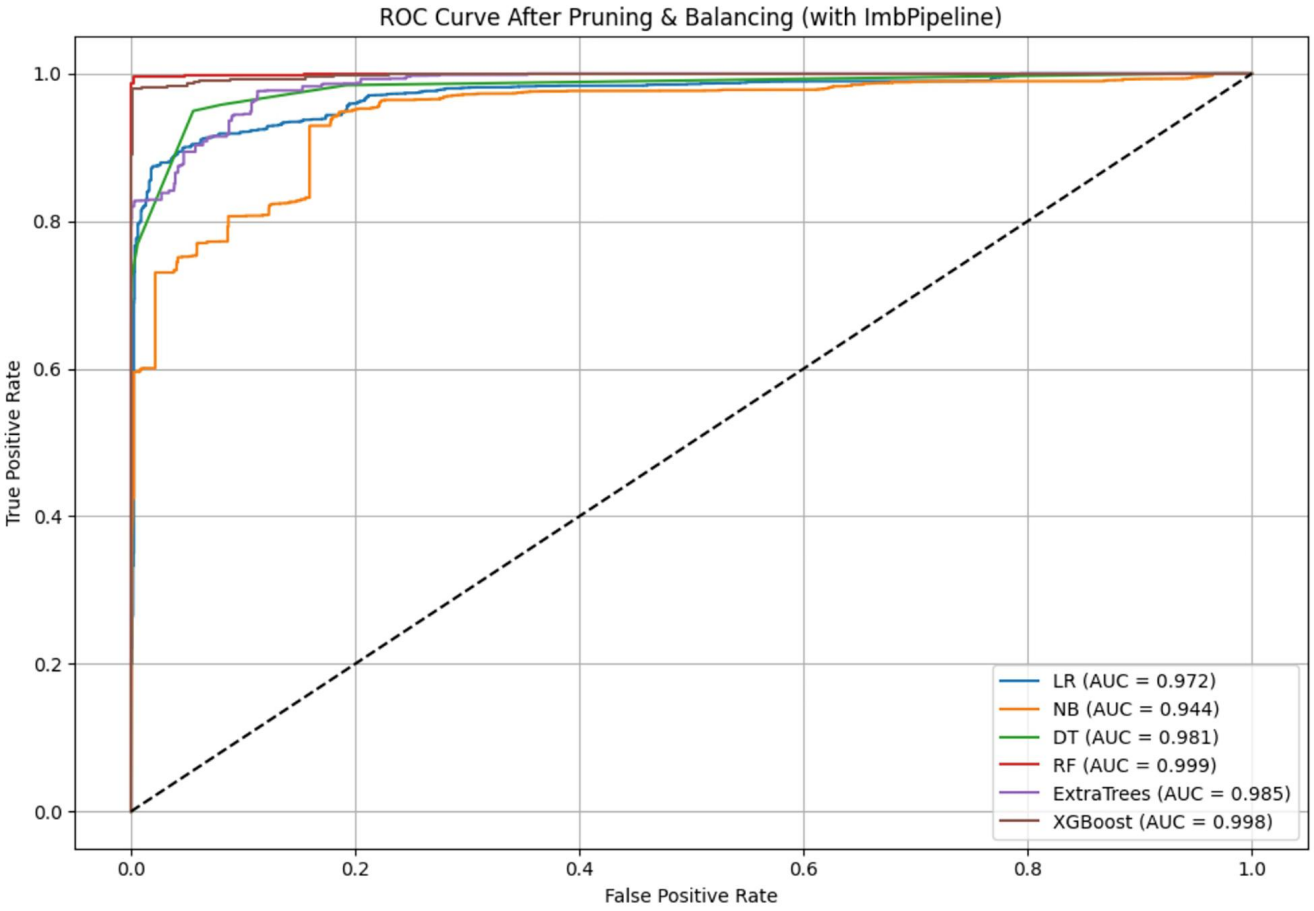
Comparison of 6 AUROC of 6 machine learning models.

**Figure 13:**
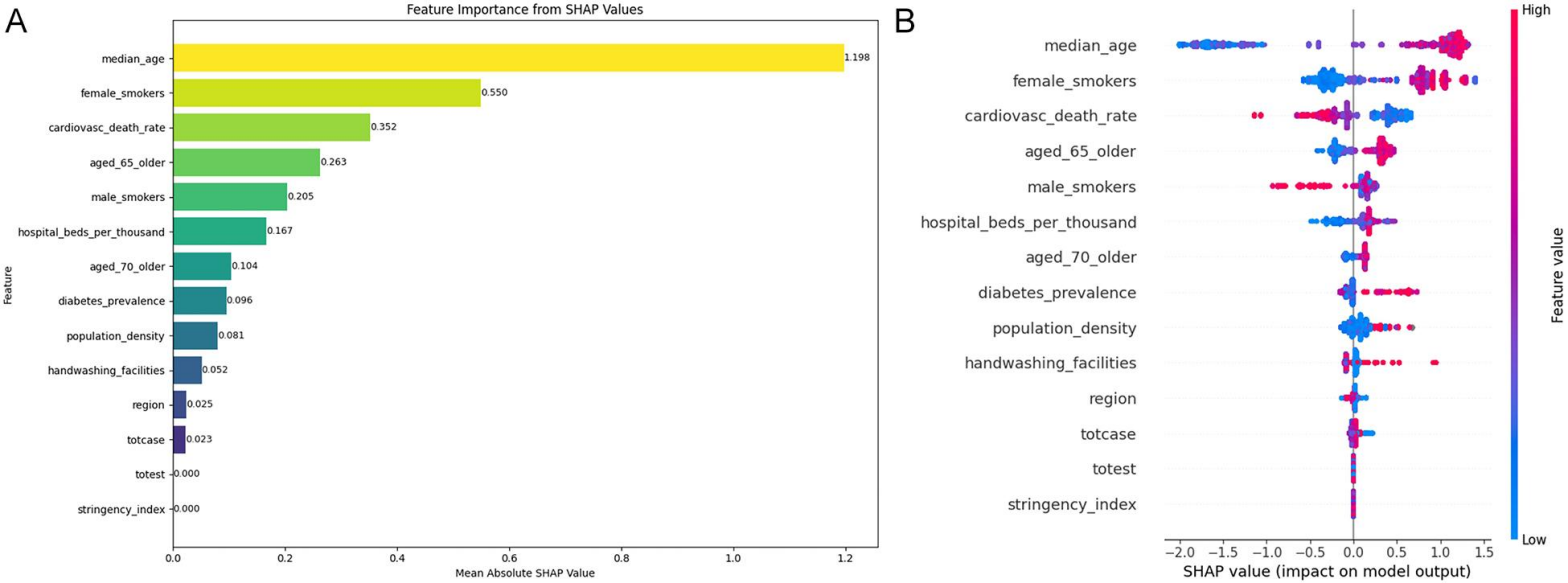
Feature importance analysis using SHAP— **(A)** SHAP bar plot and **(B)** SHAP summary plot.

Figure 11B compares six supervised machine learning algorithms—LR, DT, RF, XGB, NB, and extra trees—which are run to classify the countries in any of the two categories—“Pro-poor uptake” or “Pro-rich uptake”. 6 models are considered for final analysis and LightGBM is discarded due to its poor performance. In each of the models, the concentration score is categorized as binary (pro-rich vaccine uptake, pro-poor vaccine uptake). The same set of independent variables, i.e., geographical region and demographic, health, lifestyle, and structural determinants, are considered for the ML models. The model performance of all the models is evaluated with 3 evaluation metrics—accuracy, *F*1 score, and AUROC (Figure 11B). LR, the baseline model, had good AUROC = 0.972. This reflects low accuracy compared to the ensemble classifiers RF and XGB. NB is the lowest performer, with 81% accuracy, *F*1 score 85%, indicating a significant number of false positives and 94% explainability reflected in AUROC. DT shows strong performance with 95% accuracy, and a 96% balance in precision and sensitivity. DT, as a tree-based model, adequately considers nonlinear complexities, as evident in AUROC (0.981). RF is the best performer, with 99.1% accuracy, 99.3% balance between precision and recall, and 99.9% AUROC, overfitting is corrected after manual feature engineering, adjustment of the number of estimators, and maximum depth size. The extra trees achieved 98.5% AUROC, with 88% accuracy and 91% *F*1-score revealing low optimality compared to RF and XGB. XGB reflects 98% accuracy, and 98.7% balance in recall and precision, with 99.8% overall explainability. The weighted score ranks RF as the best model and XGB as the second-best model after eliminating the risk of overfitting. Figure 12 presents 6 (AUROC) curves for visual comparison. Figures 13A,B depicts the best performing features of the ML models. Important correlates of vaccination inequity are median age, older age, lifestyle factors, the NCD-related death rate, the prevalence of noncommunicable diseases, and the availability of basic health infrastructure.

## Discussion

This study explored the association between vaccination inequity and geographical continents—to explore how far the continents with a major share of lower-middle-income countries depict a greater degree of vaccination inequity disfavouring LICs and LMICs and how the associations react after controlling for macrolevel socioeconomic, demographic, structural, and temporal factors to investigate the prevailing nonlinearities. This study explored the utility of various supervised machine learning algorithms and identified the major correlates of inequity in access to COVID-19 vaccination—regional, socioeconomic, demographic, and structural—from the highest performing model on data from 2020 to 2023. The widening of structural inequality with economic downturns and social unrest due to stringency norms was evident in LICs and LMICs, where socioeconomic struggle is significantly greater than that in HICs (67). As shown in the present study, structural inequities are visible in front of the health system in many countries because of their weak resilience, leading to increasing socioeconomic inequities in healthcare access, which is in line with the findings from a previous study by Schnitzler et al. (68). Given this background, the current study revealed that exposure to health risk, structural, and socio-demographic correlates need robust monitoring to eradicate vaccination inequity at the global level over time.

The findings from the classical binary logistic regression models reflect the factors contributing to higher odds of pro-poor or pro-rich access to vaccination at the global level comparing different geographical regions—Africa, Asia, South America, Oceania, Europe, and North America. Model 1 reveals that, compared to high-income continents, there is significantly less pro-poor access in the African and Asian continents, indicating that coverage targeting is less pro-poor or that structural suboptimality faces challenges in tackling demand- and supply-side barriers. The scenario did not change after controlling country-level case incidence, number of tests, stringency level, demographic and socioeconomic factors from Model 2 to Model 5. This persistent pattern may partly be explained by vaccine hesitancy, which remains a significant challenge in ensuring COVID-19 vaccination coverage. Evidently, the COVID-19 vaccine acceptance rates vary within and between continents. The acceptance rate was higher in Eastern Africa than in Western Africa, mainly due to lack of trust in foreign institutions (69, 70). However, a high rate of vaccine hesitancy is evident in Eastern European countries, and policy directives were suggested to address the concerns of healthcare providers and caregivers to gain public trust in vaccine efficacy (71). In Western and Central Europe, the rate of hesitancy was significantly higher than that in some European countries, such as Ireland, Italy, Norway, and the UK, as well as in North American countries, such as Canada and the USA (69). However, regional disparities in vaccine acceptance in the USA are strongly associated with income and ethnicity (72). In Asia and the Pacific region, the vaccine acceptance rate is higher because of trust in the government; however, a high rate of vaccine hesitancy is evident in HICs such as Japan and Hong Kong (73–77). The vaccination rate is very good in Latin America and Caribbean countries, where 14 out of 20 countries have an acceptance rate greater than 70% (69).

Structural factors are found to be significant correlates to reverse vaccination inequity favouring the LICs and LMICs. The availability of basic to moderate health infrastructure was assessed using proxy indicators such as the presence of handwashing facilities in health centres and the number of hospital beds per thousand population. These indicators were used to evaluate whether health centres and hospitals possess the necessary infrastructural capacity to provide basic to emergency healthcare support. The current findings show that the availability of basic to moderate infrastructure increases the odds of pro-poor vaccine uptake in African and Asian nations, which aligns with the findings of previous studies. Vaccine rollout requires infrastructural support, such as an uninterrupted power supply for cold chains and connected supply processes, especially when immunization centres are in rural and hard-to-reach areas. However, evidence shows only 28% of health facilities in sub-Saharan Africa have a consistent energy supply (47, 78). Gray et al. (79) suggested a data-driven structural alternative for equitable delivery solutions. The distribution of vaccines based on population proportion was away from equitable vaccine distribution in comparison with the fair priority model, which indicates that countries with lower exposure to the risk of death receive vaccines at the cost of countries with higher exposure- the distribution of vaccines based on population proportions is not equitable (80). A shift from a population-based allocation to a fair-priority model was recommended for COVAX. This model prioritizes the prevention of urgent harm by focusing on saving the greatest number of expected years of life lost, without disadvantaging countries with lower life expectancy. These principles align with the findings of the present study, which show that the likelihood of pro-poor vaccination uptake increases in countries with higher burdens of NCD-related morbidity and mortality and greater exposure to unhealthy lifestyles (47, 80). Furthermore, inclusion of temporal factor shows further increase in pro-poor uptake indicating better execution of delivery model over time. However, the global average cumulative doses of COVID-19 from 2020 to 2023 manufactured by Covaxin depict enormous inequity disfavouring LICs and LMICs (Figures 1 and 2), indicating that the availability of affordable quality vaccines in LICs and LMICs requires a greater pandemic preparedness policy focus.

To explore a digital monitoring framework suitable for monitoring vaccination inequity at the global scale, 7 machine learning models are trained and tested on globally pooled data. The findings also demonstrate that ensemble learning models such as random forest and XGB significantly outperform different single learners—LR, NB, and DT—across almost all the evaluation metrics. Remarkably, the RF classifier is the best performer with highest weighted score (0.985) and XGB classifier depicts the second best overall balanced performance (0.979) compared to traditional classical logistic regression model performance (AUROC = 0.976), closely aligns with prior findings that emphasize ML models over classical models (51, 53). Different studies also have shown robust performance of ensemble tree-based classifiers in the prediction of vaccination equity using ML models (57) or in the prediction of daily COVID-19 cases (81). RF is the best model for the prediction of COVID-19 vaccine type and mortality possibility (82). Our findings support above-mentioned previous findings.

Furthermore, the findings of the present study are strongly concordant with those of one earlier study that applied ML models such as LR and DT to examine vaccine access and hesitancy and demonstrated that financial hardship due to COVID-19, suffering from chronic conditions or mental health issues, lack of access to insurance, and limited availability of testing services were consistent predictors of vaccine hesitancy among less-affluent population subgroup (83). In addition, they inferred that ensemble models outperformed logistic regression in revealing complex nonlinear patterns (83). In the present study, logistic regression, although well interpretable, performed relatively well (accuracy: 0.918, AUROC: 0.972) compared to NB, which performed relatively poorly in comparison with tree-based ensemble learning algorithms, supporting the limitations of linear model predictions in grasping the multifactorial disparities. A study by Dodoo et al. (84) exploring the barriers to vaccine uptake in Ghana revealed that RF is a better performing model than are LR and GLM. In the present study, the DT performs well (*F*1-Scores: 0.963 and AUROC: 0.981); however, as a single learner, it might have missed crucial regions in feature spaces that are efficiently considered in ensemble tree-based learning in RF and XGBoost, as also mentioned in Sarmah et al. (85), who presented scientific reasons behind the higher efficiency of ensemble learning over any single learning method in machine learning.

The current study indicates that machine learning models outperform classical predictive analysis in identifying the structural determinants of global vaccination inequity. Notably, the performance of tree-based ensemble methods has the potential to increase the efficiency of public health decision support systems with higher accuracy and precision of evidence-based policy decisions at any level of preparedness planning—global, regional, or national. Therefore, for innovations to achieve early and large-scale adoption, it is crucial to test within specific contexts before implementation. This will help assess stakeholder needs as well as the outer and inner settings, allowing interventions to be planned based on realistic scope and contextual considerations. This study presents an indicative analysis based on global-level pooled data that can be adopted in local data ecosystems, partnering with normative frameworks at the international level to ensure equitable access to vaccines.

Collectively, the evidence from current study is in line with other previous studies which indicates that addressing vaccination inequity requires multisectoral planning and strategic approaches integrating health system strengthening, targeted behavioral interventions for LICs and LMICs, and participatory policy design. Lessons from, e.g., Botswana, Nigeria, Palestine, Slovakia suggest that targeted investments in workforce towards trust-building, organized infrastructural support for effective risk coordination are critical for improving equitable vaccine uptake (41–43). Community-driven service models provide practical frameworks for reducing structural barriers and enhancing outreach among marginalized populations which can be considered while phasing the diffusion stages (46). Furthermore, research on healthcare access in the pandemic-era reinforces the urgency of resilient immunization systems to reduce global disparities in access mitigating any structural disruptions (41).

## Conclusion

The COVID-19 pandemic impaired the global health systems resilience, intensified and widened disparities in access. This study proposes an artificial intelligence-driven monitoring framework using conventional statistical models and machine learning on vaccine uptake data at global level. Findings reveal distribution of getting vaccinated is largely pro-rich in Africa, North America, Europe, and Oceania, while Southeast Asia shows pro-poor uptake. Controlling for structural determinants, health system capacity, hygiene infrastructure, and risk prioritization especially in LMICs where risk structures are complex significantly increases the likelihood of equitable vaccine access in African and Asian low resource settings over time. Among the estimated models used for classification, Random Forest and XGBoost achieved the highest weighted score (98.5% and 97.9% respectively), emphasizing the effectiveness of ensemble machine learning for monitoring vaccination inequity.

### Limitations

Despite these findings, the current study has several limitations. The study considered multiple secondary datasets and relied on aggregated indicators at the country level. Country-level aggregation and averaging inherently lead to the loss of multilevel variabilities considering all levels of health administrative hierarchies and layers within each microlevel community unit, which consists of community-, household-, and individual-level variabilities that may hide between and within-cluster heterogeneities, thereby weakening the precision of statistical inference.

Furthermore, datasets collected using various methodologies and reporting benchmarks raise concerns about data synchronization and comparability. This may contribute to measurement bias and inconsistent results. Such possibilities are minimized by considering well-known country-level indicators from the WHO and World Bank databases to avoid any ambiguity. Additionally, the use of national-level aggregates suppresses individual-level attributes; thus, inferences are made at the macro level, which are not recommended for microlevel generalizations. Although the main dataset used covers the daily data, no causality is explored, limiting the ability of the study to draw causal inference. Finally, as the theoretical framework includes a few socioeconomic, demographic, and lifestyle factors aggregated at the national level, unobserved confounding factors—the influence of omitted variables on both covariates and outcome—cannot be ruled out. The models are interpreted very carefully, keeping in mind the limitations to indicate policy directions.

### Novelty

On the other hand, this study depicts uniqueness with a blend of methodological advancements under the umbrella of health analytics. First, it combines classical models with ML classifiers to classify COVID-19 vaccine inequity across 195 countries, which is applied to other health issues before but significant application with respect to COVID-19 vaccination inequity was less evident. Second, it leverages a large-scale dataset integrated from multiple sources to apply the abovementioned modelling approach, which, to the best of the researcher's knowledge, has not previously been considered for comprehensive analysis in health equity research to inform global policy. Third, the study links a classical normative health economics measure, the concentration index, with cutting-edge predictive analytics. This integration strengthens health economics tools by incorporating ensemble learning and proposes a digital monitoring framework capable of estimating inequity in real time for future unseen data. Finally, it has provided a digital health economic solution to make COVID-19 preparedness smart and intelligent for efficient targeting in LICs and LMICs.

### Future research directions

**Figure 14:**
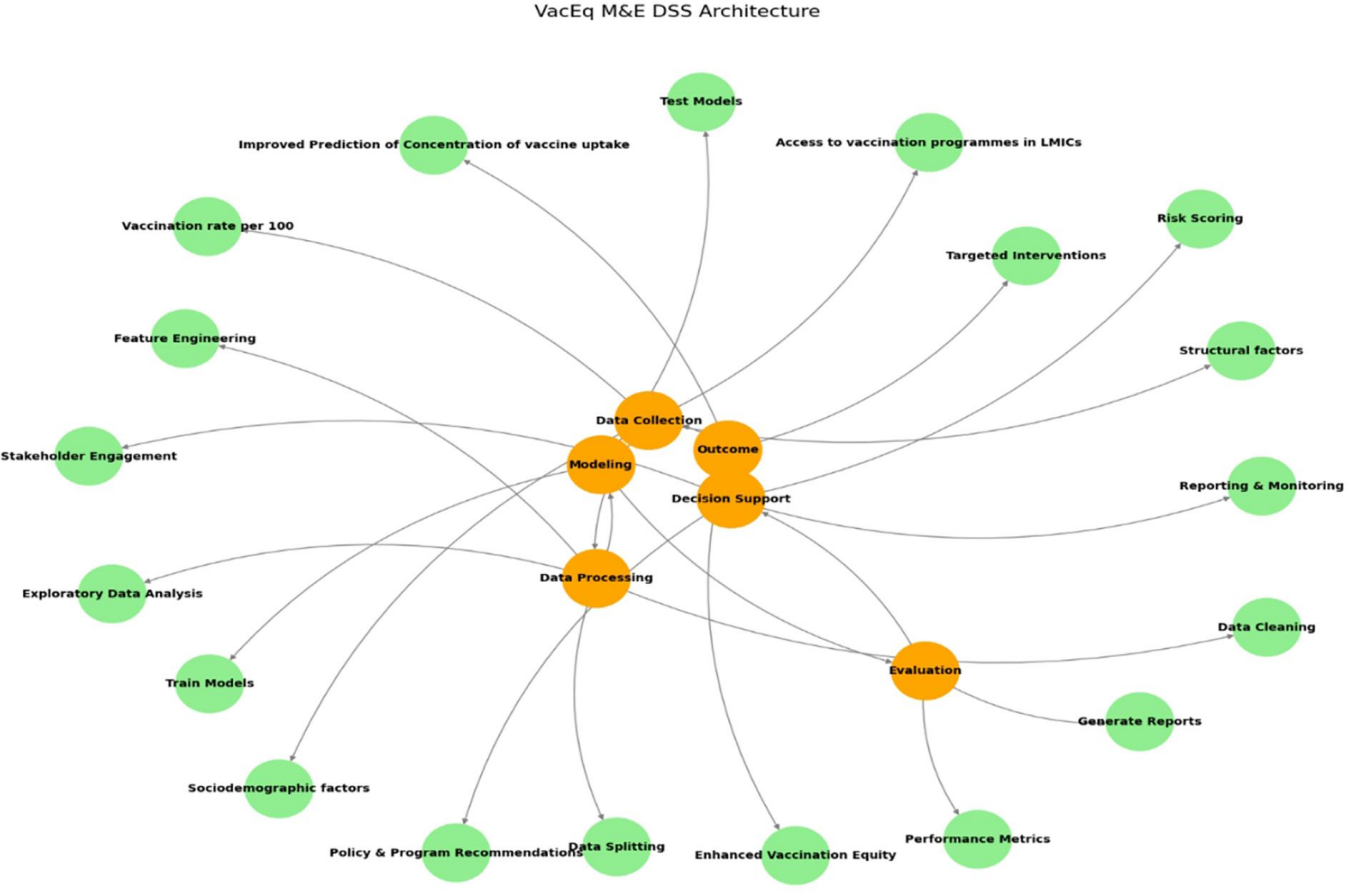
Vaccination equity M&E DSS architecture.

Future research should focus on developing this concept in the global-regional-national mode with community participation. Sustainable early adoption of such digital health economic interventions to monitor and address inequities for preparedness planning can be achieved only with effective piloting to prevent future pandemics. In addition, subtle challenges related to structural barriers in the diffusion of digital innovation should be tested in resource-scarce settings where the higher computational cost of complex ensemble learning algorithms limits the early adoption of these models. Therefore, piloting the prototype in a local setting to explore social, economic, temporal, operational, technical, and ethical feasibility can cocreate acceptable and usable solutions. An example of a decision support architecture for ensuring vaccination equity is presented in Figure 14.

In summary, structural factors determine the success of access-related interventions in ensuring equity. Despite these barriers, the pandemic response has shown the usability and effectiveness of digital health interventions in contact tracing, outbreak analysis and prediction, and monitoring of repeat services enhances the efficiency of health systems (86). Global health security and “health for all” mandates a focus on addressing multifaceted challenges in LICs and LMICs to reduce the health disparities and inequities in access aggravated by the COVID-19 pandemic. The new SPRP objectives emphasize evidence-informed policy decisions fostering solidarity-based coordination and international collaboration. To make policy decisions evidence-based, an integrated data-driven intelligent public health system from the global to the local level is needed. The current research was built on this advancement, explored whether structural factors are associated with inequitable COVID-19 vaccine access and proposed an AI-driven monitoring framework to support digital transformation in pandemic preparedness initiatives for LICs and LMICs from a health economic lens. The current study attempted to apply better predictive models to analyse routine monitoring data on vaccine uptake with the application of machine learning. This step has increased predictive accuracy to help policy decisions identify location-specific moderating and mediating factors and reduce the levels of vaccination inequity as visible in different other applications of machine learning in healthcare to improve policy directives (87–89). Furthermore, it is expected to increase social benefits from government spending, ensuring efficiency-led equity in the health sector to correct market failure and achieve market equilibrium, ensuring survival and quality of life.

## Data Availability

The original contributions presented in the study are included in the article/Supplementary Material, further inquiries can be directed to the corresponding author.
